# The effect of an orally‐dosed *Gynostemma pentaphyllum* extract (ActivAMP®) on body composition in overweight, adult men and women: A double‐blind, randomised, placebo‐controlled study

**DOI:** 10.1111/jhn.12936

**Published:** 2021-08-09

**Authors:** Amanda Rao, Paul Clayton, David Briskey

**Affiliations:** ^1^ RDC Clinical Brisbane QLD Australia; ^2^ School of Medicine University of Sydney Sydney NSW Australia; ^3^ Institute of Food, Brain and Behaviour Oxford UK; ^4^ School of Human Movement and Nutrition Sciences University of Queensland Brisbane QLD Australia

**Keywords:** ActivAMP®, body composition, body weight, *Gynostemma pentaphyllum*, herbal extract

## Abstract

**Background:**

The present study examined the effect of a herbal supplement containing a *Gynostemma pentaphyllum* (Gpp) extract (ActivAMP®) with respect to improving body composition in overweight males and females.

**Methods:**

One‐hundred and seventeen men and women aged over 18 years completed 16 weeks of daily supplementation with either Gpp or a placebo. Participants underwent dual‐energy X‐rays to assess body composition (fat mass, lean mass and mass distribution), as well as anthropometric measures (weight, height, hip and waist circumference), in addition to blood tests to assess inflammatory and safety markers.

**Results:**

Following 16 weeks of treatment, the Gpp group had a significant reduction in total body weight, body mass index, total fat mass and gynoid fat mass compared to the placebo group. Blood measures showed plasma triglyceride, alanine aminotransferase and tumour necrosis factor‐α to be statistically different between groups at week 16. Subgroup analysis of gender for fat distribution showed males in the Gpp group had a significant reduction in visceral fat compared to males in the placebo group and females in the Gpp group had a significant reduction in gynoid fat compared to the placebo group.

**Conclusions:**

Gpp was capable of altering fat mass and fat distribution in overweight and obese males and females compared to a placebo.

## Graphical abstract

The therapeutic properties of *Gynostemma pentaphyllum* are proposed to be related to activation of AMP‐kinase and there is a growing body of evidence to support its use in weight loss. The results of this double‐blind randomised placebo‐controlled trial in overweight men and women indicate that supplementation with *G. pentaphyllum* over 16 weeks reduced body weight and fat mass.

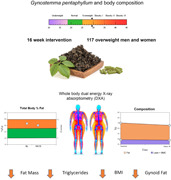

## INTRODUCTION

Obesity represents a major health and societal concern worldwide. Already prevalent in high‐income countries, the prevalence of obesity is now increasing in low and middle‐income countries, driven by multiple factors, including the post‐transitional diet.^1^, ^2^ Obesity significantly increases the risk of type 2 diabetes,^3^ cardiovascular disease, stroke, renal and hepatic disease, and a range of cancers.^4^, ^5^, ^6^ Current interventions for obesity have poor compliance and poor efficacy as evidenced by the growing obesity pandemic. There is a need for interventions that are effective and safe for personal use and integration into public health strategies.

Traditional treatments for obesity derived from natural compounds show great promise in the management of obesity. One such compound is *Gynostemma pentaphyllum*, a herbaceous climbing vine belonging to the Cucurbitaceae family (cucumber or gourd family).^7^ This herb has been widely used in East Asian countries as a traditional herbal tea and medicine to treat obesity and diabetes,^8^ amongst other diseases. Partly as a result of its documented anti‐diabetic activity,^8^, ^9^, ^10^, ^11^ it is routinely used today in China as a treatment for hyperlipidaemia, fatty liver and obesity.^12^

The therapeutic activities of *G. pentaphyllum* are proposed to be related to the AMP‐activated protein kinase (AMPK)‐activating effects of the dammarane‐type saponins.^13^, ^14^, ^15^ The mechanism of action of dammaranes, which involves the activation (phosphorylation) of AMPK,^13^, ^14^, ^15^ mimics the effects of physical exercise, including an up‐regulation of autophagy^16^, ^17^ with increased mitochondrial neogenesis^18^ and glucose transporter‐4 expression,^19^ as well as a subsequent improvement of insulin sensitivity.^20^ AMPK, an intracellular energy sensor and key regulator of metabolic homeostasis, is an emerging target for metabolic diseases such as obesity and diabetes.^21^ AMPK increases ATP production and decreases energy consumption by responding to cellular stress.^13^ Because AMPK is activated in conditions that increase the intracellular AMP:ATP ratio, such as exercise or starvation,^22^ *G. pentaphyllum* represents a potential treatment for obesity management that can complement lifestyle modifications such as exercise and diet restriction. Given the large and growing problems of overweight, physical inactivity, insulin resistance and non‐alcoholic fatty liver disease (NAFLD),^16^, ^23^ *G. pentaphyllum* has a potentially important role to play in improving this key aspect of public health.

Existing safety data,^24^, ^25^ the absence of toxicity reported in clinical trials^26^, ^27^, ^28^ and the growing body of evidence that *G. pentaphyllum* supports weight loss^12^, ^13^, ^14^, ^26^, ^27^ provide a rationale for developing this traditional remedy as a public health tool in the management of overweight and obesity. Therefore, the present study aimed to assess the efficacy and safety of a commercially available capsule‐form herbal supplement containing *G. pentaphyllum* extract (ActivAMP®; Gencor Lifestage Solutions) with respect to improving body composition in overweight males and females.

## METHODS

A double‐blind, randomised, clinical trial with a treatment duration of 16 weeks, including ActivAMP®, a commercially available capsule‐form herbal supplement containing *G. pentaphyllum* ([Gpp], supplied by BTC Corporation, Korea) and placebo groups. Participants who met the preliminary screening criteria via a telephone interview underwent full screening against the inclusion and exclusion criteria (detailed below), which included collecting lifestyle, current medications and medical history. Eligible participants provided written consent for enrollment and once enrolled were randomly allocated to either the Gpp or placebo intervention group via Random Allocation Software (www.sealedenvelope.com) conducted by an individual who was not involved in the trial.

Following enrollment, participants completed baseline measures, which included body composition (height, weight, hip and waist circumference), dietary intake and quality of life. Participants were then referred to a pathology clinic to provide a blood sample and medical imaging centre for a dual‐energy X‐ray absorptiometry (DXA) assessment for body composition (total body fat and fat‐free mass).

During the 16‐week intervention trial, participants were required to attend the study clinic at weeks 5 and 10 for anthropometry assessment and to complete questionnaires. Every 2–3 weeks, participants completed 24‐h diet recalls via online diaries and through telephone interviews conducted by a trial investigator. At trial completion (16 weeks), an assessment identical to that undertaken at baseline was conducted.

Participants were asked to maintain their usual level of physical activity and dietary intake for the study duration. At the end of the study, participants repeated the same exercise and diet diaries. If participants knowingly changed their normal level of physical activity or diet, they were asked to inform a trial investigator as soon as practically possible. Changes in diet and/or exercise were evaluated by a dietitian and accredited exercise physiologist respectively. Any changes to diet or exercise were taken into consideration when evaluating any results of the trial. Participants were also monitored for compliance with the protocol by email communications in addition to each scheduled site visit.

All study participants were recruited from Brisbane and surrounding areas from databases and public media outlets. The inclusion criteria included: males and females over 18 years of age who were overweight and class one obese (body mass index [BMI] > 25 to < 35 kg m^–2^). Other inclusion criteria included not currently taking any supplements or functional foods targeted at weight loss, muscle growth or exercise performance, agreeing to not use other treatments including diets for weight loss, muscle growth or exercise performance during the study, and having the ability and willingness to participate in the study and adhere to the investigation schedule. Only females currently using an appropriate form of birth control (e.g. oral contraceptive pill) were included in the study. Participants were excluded from the study if they had any clinically significant medical condition that was uncontrolled, including, but not limited to, cardiovascular, neurological, psychiatric, renal, gastrointestinal, immunological, endocrine (including uncontrolled diabetes or thyroid disease) or haematological abnormalities. Participants were also excluded if they used prescription medication (other than the oral contraceptive pill), had significant variation in weight (more than 10%) in the past 3 months, participated in another clinical trial in the past 3 months, were allergic or hypersensitive to any of the test ingredients, consumed alcohol above two standard drinks daily, used recreational drugs or had other confounding conditions as assessed by trial investigators. Females with a clinical diagnosis of polycystic ovarian syndrome, attempting conception or were currently pregnant or breast‐feeding were excluded from the study. In total, 150 participants met the criteria and were randomly divided into two groups: Gpp (*n* = 75) or Placebo (*n* = 75).

Participants consumed 450 mg of either Gpp or a placebo with water across two capsules (225 mg per capsule) daily, one taken at breakfast and one taken at dinner for a period of 16 weeks. This regime was selected on the basis of current standard dosing guidelines for the investigational product. The placebo product consisted of maltodextrin housed in an opaque gel capsule identical to the test product.

The primary outcome was total body fat as measured by a DXA scan. Secondary outcomes included lean and fat mass distribution (from DXA), BMI, body weight, hip and waist circumferences, plasma measures (total cholesterol, triglycerides, low‐density lipoprotein‐cholesterol, high‐density lipoprotein‐cholesterol, free fatty acids, blood glucose, kidney function/safety, plasma liver function/safety, peripheral blood mononuclear cells and an inflammatory panel) and participant's quality of life [36‐Item Short Form Survey (SF‐36)] and dietary intake.

Sample size was calculated using G*power, version 3.0.10 (http://www.gpower.hhu.de). Accounting for an α error probability of 0.05 and powered to 0.95 for a 5% change in body composition (from baseline values; DXA; effect size *d* = 0.72), using the mean (30% body fat) and SD values (±5.5%) obtained from similar published studies, group sizes of at least 42 were required. However, allowing for an approximately 40% dropout rate, group sizes were set as 75 each (*n* = 150 total).

Data were analysed with R (R Foundation for Statistical Computing), using a range of native statistical functions and functions from the packages tidyverse, rcompanion, dplyr and ggplot. Shapiro–Wilks tested for normality of distribution, tests of significance were performed both parametrically and non‐parametrically. Some variables have a log‐normal distribution and were logN transformed for *t* test analysis. *p* values for *t* tests or Mann–Whitney *U* tests showing likelihood of difference between the listed Gpp and placebo were considered statistically significant at *p* < 0.05.

The trial was conducted in compliance with the current International Conference on Harmonization Guideline for Good Clinical Practice. The study protocols were approved by Bellberry Ltd Human Research and Ethics Committee (2016‐11‐832) and listed on the Australian and New Zealand Clinical Trial Register (ACTRN12617000839303).

## RESULTS

**Figure 1:**
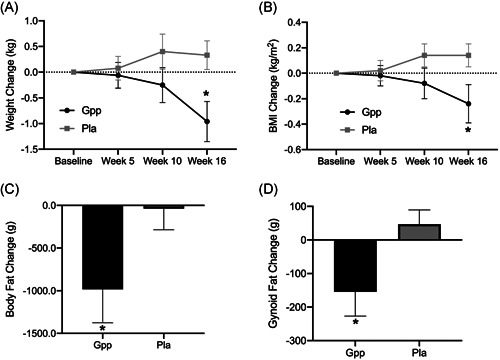
Body composition data. Body weight and dual‐energy X‐ray absorptiometry (52 participants per group). (A) Weight change from baseline. (B) BMI change from baseline. (C) Body fat change from baseline. (D) Gynoid fat change from baseline. BMI, body mass index; Gpp, *Gynostemma pentaphyllum*; Pla, placebo

Of the 150 participants enrolled in the study, 117 completed the study (60 for Gpp and 57 for placebo) (Figure 1). Of the 117 completed participants, full sets of DXA results were collected for 104 participants (*n* = 52 per group) and full sets of blood results were collected for 104 participants (56 for Gpp and 48 for placebo). There were no statistical differences between groups at baseline (Table 1).

**Table 1:** Participant details

	Gpp	Placebo
Randomised (*n*)	75	75
Completed (*n*)	60	57
Female (*n*)	33	30
Male (*n*)	27	27
Withdrawn (total) (*n*)	15	18
Withdrawn (did not complete baseline) (*n*)	8	10
Age mean (SD) (years)	49.4 (13.3)	50.7 (10.4)

Abbreviation: Gpp, *Gynostemma pentaphyllum*.

After 16 weeks of treatment, the Gpp group had a significant reduction in total body weight, BMI, total fat mass and gynoid fat mass compared to the placebo group (*p* < 0.05) (Figure 1 and Table 2). Percentage fat mass decreased (1.2%) in the Gpp group, which was trending toward significance (*p* = 0.08). There were no significant differences for waist or hip circumference between groups (Table 2).

**Table 2:** Body composition

	Baseline		Δ Week 16		*p* value*
	Gpp	Placebo	Gpp	Placebo	
Body mass (kg)	88.1 (16.7)	89.2 (13.8)	−0.96	0.31	0.02*
Lean mass (kg)	54.9 (14.5)	55.5 (12.4)	0.12	0.33	0.480
Fat mass (kg)	30.5 (6.9)	31.2 (7.5)	−0.98	−0.04	0.048*
Lean mass (%)	61.8 (7.0)	61.8 (7.6)	0.73	0.13	0.07
Fat mass (%)	35.1 (7.2)	35.3 (7.8)	−1.26	−0.10	0.09
Fat mass trunk (kg)	15.7 (3.9)	15.8 (4.6)	−0.67	−0.12	0.08
Body mass index (kg m^–2^)	29.6 (3.3)	30.4 (3.1)	−0.24	0.14	0.049*
Android mass (kg)	2.8 (0.9)	2.9 (1.0)	−0.15	−0.03	0.07
Gynoid mass (kg)	5.1 (1.5)	5.1 (1.7)	−0.16	0.05	0.03*
Android/gynoid ratio	1.1 (0.2)	1.1 (0.2)	−0.026	−0.008	0.08
Visceral fat (g)^a^	854.4 (590.0)	818.7 (597)	−36.7	7.19	0.22
Waist circumference (cm)	100.4 (11.6)	101.4 (10.1)	−0.46	−0.39	0.90
Hip circumference (cm)	113.3 (7.1)	113.0 (7.7)	−0.79	−0.48	0.30

*Note:* Dual‐energy X‐ray absorptiometry data collected for 52 participants from both groups.

Abbreviations: Gpp, *Gynostemma pentaphyllum*; Δ, change from baseline.

^a^ These variables have a log‐normal distribution and were logN transformed for *t*‐test analysis.

* *p* values for likelihood of difference between the Gpp and placebo groups.

There was no difference between groups for any of the blood markers at baseline. After 16 weeks of treatment, plasma triglyceride, alanine aminotransferase and tumour necrosis factor (TNF)‐α were statistically different between groups (*p* < 0.05) (Table 3).

**Table 3:** Pathology results

	Baseline		Week 16		
	Gpp	Placebo	Gpp	Placebo	*p*‐values
Alanine aminotransferase (U L^–1^)^a^	28.3 (15.7)	31.7 (18.1)	27.4 (11.4)	33.6 (19.2)	0.03*
Aspartate aminotransferase (U L^–1^)^b^	27.7 (9.5)	30.7 (13.8)	28.7 (8.4)	31.2 (13.0)	0.24
Gamma‐glutamyl transferase (U L^–1^)^a^	30.6 (22.8)	28.6 (13.0)	29.6 (22.3)	27.7 (13.5)	0.84
Cholesterol (mmol L^–1^)	5.4 (1.0)	5.7 (1.0)	5.4 (1.1)	5.6 (1.0)	0.15
High‐density lipoprotein (mmol L^–1^)^a^	1.5 (0.4)	1.6 (0.6)	1.58 (0.4)	1.60 (0.6)	0.57
Low‐density lipoprotein (mmol L^–1^)	3.1 (0.9)	3.5 (0.9)	3.16 (1.0)	3.37 (1.0)	0.14
Triglycerides (mmol L^–1^)^a^	1.21 (0.8)	1.42 (0.8)	1.09 (0.5)	1.35 (0.7)	0.02*
Bilirubin (µmol L^–1^)^a^	11.3 (4.5)	12.0 (4.5)	13.1 (10.1)	11.7 (4.4)	0.56
Glucose (mmol L^–1^)^b^	5.4 (1.1)	5.3 (2.7)	5.3 (1.0)	5.3 (0.7)	0.34
Insulin (µU mL^–1^)^a^	11.3 (7.9)	10.9 (7.0)	10.6 (7.6)	11.9 (8.2)	0.24
Tumour necrosis factor‐α (pg mL^–1^)	9.3 (1.5)	9.7 (2.3)	8.2 (1.7)	10.0 (2.2)	0.018*
Interleukin‐10 (pg mL^–1^)	11.7 (7.6)	11.8 (8.8)	10.7 (7.1)	13.2 (7.2)	0.42
Interleukin‐8 (pg mL^–1^)	24.1 (26.2)	28.2 (34.6)	23.2 (24.2)	29.5 (37.5)	0.6

*Note:* Pre‐ and post‐pathology data was available for 56 participants in the Gpp group and 48 in the placebo group.

Abbreviation: Gpp, *Gynostemma pentaphyllum*.

^a^ These variables have a log‐normal distribution and were logN transformed for *t* test analysis; *p* values for *t* tests or Mann–Whitney *U* tests showing likelihood of difference between groups.

^b^ Shapiro–Wilks distribution test found these data to be not normally distributed; tests of significance are performed non‐parametrically.

* *p* < 0.05.

There was no significant difference at baseline or week 16 for the SF‐36 health questionnaire between groups. The results indicated that all participants had good general health. Throughout the study, there was no change to any participants exercise or diet habit. Seven participants reported adverse events during the study: five in the Gpp treatment group (diarrhoea, insomnia, dizziness, nausea, dry throat) and two in the placebo group (fatigue and increased bowel movements).

Subgroup analysis of gender for android, gynoid and visceral adiposity showed males in the Gpp group had a significant reduction in visceral fat compared to males in the placebo group (−109 vs. 12 g; *p* < 0.05). Females in the Gpp group had a significant reduction in gynoid fat compared to the placebo group (−107 vs. 95 g; *p* < 0.05). No subgroup differences were seen for android fat.

## DISCUSSION

The present study assessed the efficacy of *G. pentaphyllum* (ActivAMP®) on body composition (including fat mass, lean mass and fat mass distribution) as measured by DXA in overweight men and women aged over 18 years of age. Secondary outcomes included BMI, body weight, hip and waist circumferences, plasma measures, quality of life (SF‐36), and dietary intake.

The outcomes of this trial support earlier findings of the clinical effectiveness of *G. pentaphyllum* with respect to supporting weight loss, with good tolerability.^26^, ^27^, ^28^ Furthermore, these results extend the case for *G. pentaphyllum* as an aide to weight management. Although prior work focused on the overweight (BMI < 30 kg m^–2^) population, the present trial included class 1 obese participants.

Following 16 weeks of supplementation, the Gpp group had a significant reduction in total body weight, fat mass and BMI compared to the placebo group. Subgroup analysis for gender differences showed that this effect was largely a result of males having a significant reduction in visceral fat (abdominal), whereas females had a significant reduction in gynoid fat (hips and thighs), compared to the placebo group. This is plausible because males tend to carry fat predominantly around the abdominal region and females around the hips and thighs.^29^, ^30^ In the case of males especially, targeting abdominal fat may also help prevent diseases linked to a higher waist circumference.

Despite the observed reduction in body fat, there was no reduction in waist or hip circumference between groups. It may be that a greater weight loss is required to achieve sufficient change in the waist or hip circumference to achieve a significant difference. Therefore, a longer trial period in future studies may be required to see a change in these parameters. Another factor may the mixed genders in the study. With the previously discussed differences in location of fat mass loss, this would likely translate into differences in changes to waist and hip circumferences. Therefore, the males may be more likely to have a reduction in waist circumference and females a reduction in hip circumference. By conducting a mixed gender study, a possible effect on hip or waist circumference may be diluted by the opposite gender. Although subgroup analysis for gender was conducted for waist and hip circumferences, no significant changes were seen between groups. This is likely a result of the number of participants analysed in each group. Therefore, future studies wanting to look more specifically at waist and hip circumference would benefit by having power for separate groups of males and females.

Additional observations from the present study included significant reductions in TNF‐α concentrations, which would be expected to confer an anti‐inflammatory effect. Specifically, TNF‐α levels were slightly elevated at baseline for both groups with the Gpp group returning to close to normal levels.^31^ This could have been a result of the reduction in fat mass in the Gpp group because TNF‐α levels correlate with BMI.^32^ Adipose tissue contains a significant stromal vascular fraction, which includes numerous cell types known to produce TNF‐α.^33^, ^34^, ^35^ Adipose tissue in obese individuals presents with increased infiltration of macrophages,^33^, ^34^, ^36^, ^37^ resulting in elevated production of TNF‐α.^33^ Reducing the amount of adipose tissue would therefore be expected to lower TNF‐α production. However, the present study was not designed to discriminate between the primary anti‐inflammatory effects induced by the polyphenol constituents of the Gpp and the secondary effects attributable to the reduction of adipose tissue. This will be a focus of future research.

Gpp may also be useful in chronic conditions where energy imbalance is centrally involved such as NAFLD, the eighth most common cause of death globally.^38^ Physical exercise has been proven effective as a treatment for NAFLD,^39^ with its impact mediated via the up‐regulation of autophagy.^16^, ^17^, ^40^ The ability of Gpp to induce AMP‐kinase activation and therefore autophagy,^32^, ^41^ together with its anti‐inflammatory effect, should be reviewed in this context. It also should be noted that, although autophagy may play a role in NAFLD, the full aetiology of NAFLD is complex and still to be established.^42^ Therefore, the role of Gpp in NAFLD would need to be directly tested in NAFLD to know its efficacy.

Pre‐clinical studies have already demonstrated Gpp's hepatoprotective effects in the CDDA,^43^ MCD^44^ and HFD^45^ rodent models and there is preliminary clinical data showing that Gpp plus diet is more effective than diet alone in subjects with NAFLD.^46^ This data set, together with our findings, suggests that Gpp will be a useful adjunct to physical exercise in the clinical management of NAFLD. In cases where exercise is contra‐indicated or impossible, Gpp may also have value as a stand‐alone in the management of this otherwise intractable condition.

In conclusion, *G. pentaphyllum* appears to be a promising compound capable of altering fat mass and fat distribution in overweight and obese males and females.

## AUTHOR CONTRIBUTIONS

*Conceptualisation*: Amanda Rao and David Briskey. *Methodology*, Amanda Rao and David Briskey. *Analysis*, Amanda Rao. *Writing – original draft preparation*, Paul Clayton and David Briskey. *Writing – review and editing*, Amanda Rao, Paul Clayton and David Briskey. All authors have read and agreed to the final version of the manuscript submitted for publication.

## CONFLICT OF INTERESTS

The authors declare that there are no conflict of interests.

## References

[1] Popkin BM . The nutrition transition in low‐income countries: an emerging crisis. Nutr Rev. 1994;52:285–98. 7984344 10.1111/j.1753-4887.1994.tb01460.x

[2] Huang L , Wang Z , Wang H , Zhao L , Jiang H , Zhang B , et al. Nutrition transition and related health challenges over decades in China. Eur J Clin Nutr. 2021;75:247–52. 32620907 10.1038/s41430-020-0674-8

[3] Colditz GA , Willett WC , Rotnitzky A , Manson JE . Weight gain as a risk factor for clinical diabetes mellitus in women. Ann Intern Med. 1995;122:481–6. 7872581 10.7326/0003-4819-122-7-199504010-00001

[4] Managing overweight and obesity in adults: systematic review from the obesity expert panel. 2013 [cited 10 February 2021]. Available from: https://www.nhlbi.nih.gov/health-topics/managing-overweight-obesity-in-adults

[5] Clinical guidelines on the identification, evaluation, and treatment of overweight and obesity in adults – The evidence report. National Institutes of Health. Obes Res. 1998;6(Suppl 2):51S–209S. 9813653

[6] Bhaskaran K , Douglas I , Forbes H , dos‐Santos‐Silva I , Leon DA , Smeeth L . Body‐mass index and risk of 22 specific cancers: a population‐based cohort study of 5·24 million UK adults. Lancet. 2014;384:755–65. 25129328 10.1016/S0140-6736(14)60892-8 PMC4151483

[7] Shi Y , Zou R , Liu B . Complete chloroplast genome sequence of *Gynostemma pentaphyllum* (Cucurbitaceae), a perennial medicinal herb. Mitochondrial DNA B Resour. 2019;4:3967–8. 33366273 10.1080/23802359.2019.1688726 PMC7707782

[8] Lokman EF , Gu HF , Wan Mohamud WN , Östenson CG . Evaluation of antidiabetic effects of the traditional medicinal plant *Gynostemma pentaphyllum* and the possible mechanisms of insulin release. Evid Based Complement Alternat Med. 2015;2015:120572. 26199630 10.1155/2015/120572 PMC4493304

[9] Hoa NK , Phan DV , Thuan ND , Ostenson CG . Screening of the hypoglycemic effect of eight Vietnamese herbal drugs. Methods Find Exp Clin Pharmacol. 2009;31:165–9. 19536359 10.1358/mf.2009.31.3.1362514

[10] Norberg A , Hoa NK , Liepinsh E , Van Phan D , Thuan ND , Jörnvall H , et al. A novel insulin‐releasing substance, phanoside, from the plant *Gynostemma pentaphyllum* . J Biol Chem. 2004;279:41361–7. 15220351 10.1074/jbc.M403435200

[11] Hoa NK , Norberg A , Sillard R , Van Phan D , Thuan ND , Dzung DT , et al. The possible mechanisms by which phanoside stimulates insulin secretion from rat islets. J Endocrinol. 2007;192:389–94. 17283239 10.1677/joe.1.06948

[12] Wang M , Wang F , Wang Y , Ma X , Zhao M , Zhao C . Metabonomics study of the therapeutic mechanism of *Gynostemma pentaphyllum* and atorvastatin for hyperlipidemia in rats. PLoS One. 2013;8:e78731. 24223845 10.1371/journal.pone.0078731 PMC3815346

[13] Nguyen PH , Gauhar R , Hwang SL , Dao TT , Park DC , Kim JE , et al. New dammarane‐type glucosides as potential activators of AMP‐activated protein kinase (AMPK) from *Gynostemma pentaphyllum* . Bioorg Med Chem. 2011;19:6254–60. 21978948 10.1016/j.bmc.2011.09.013

[14] Gauhar R , Hwang SL , Jeong SS , Kim JE , Song H , Park DC , et al. Heat‐processed *Gynostemma pentaphyllum* extract improves obesity in ob/ob mice by activating AMP‐activated protein kinase. Biotechnol Lett. 2012;34:1607–16. 22576281 10.1007/s10529-012-0944-1

[15] Cool B , Zinker B , Chiou W , Kifle L , Cao N , Perham M , et al. Identification and characterization of a small molecule AMPK activator that treats key components of type 2 diabetes and the metabolic syndrome. Cell Metab. 2006;3:403–16. 16753576 10.1016/j.cmet.2006.05.005

[16] Chun SK , Lee S , Yang MJ , Leeuwenburgh C , Kim JS . Exercise‐induced autophagy in fatty liver disease. Exerc Sport Sci Rev. 2017;45:181–6. 28419000 10.1249/JES.0000000000000116 PMC5479347

[17] He C , Bassik MC , Moresi V , Sun K , Wei Y , Zou Z , et al. Exercise‐induced BCL2‐regulated autophagy is required for muscle glucose homeostasis. Nature. 2012;481:511–5. 22258505 10.1038/nature10758 PMC3518436

[18] Reznick RM , Shulman GI . The role of AMP‐activated protein kinase in mitochondrial biogenesis. J Physiol. 2006;574:33–9. 16709637 10.1113/jphysiol.2006.109512 PMC1817787

[19] Koh JH , Hancock CR , Han DH , Holloszy JO , Nair KS , Dasari S . AMPK and PPARβ positive feedback loop regulates endurance exercise training‐mediated GLUT4 expression in skeletal muscle. Am J Physiol Endocrinol Metab. 2019;316:E931–9. 30888859 10.1152/ajpendo.00460.2018 PMC6580175

[20] Borghouts LB , Keizer HA . Exercise and insulin sensitivity: a review. Int J Sports Med. 2000;21:1–12. 10683091 10.1055/s-2000-8847

[21] Zhang BB , Zhou G , Li C . AMPK: an emerging drug target for diabetes and the metabolic syndrome. Cell Metab. 2009;9:407–16. 19416711 10.1016/j.cmet.2009.03.012

[22] Herzig S , Shaw RJ . AMPK: guardian of metabolism and mitochondrial homeostasis. Nat Rev Mol Cell Biol. 2018;19:121–35. 28974774 10.1038/nrm.2017.95 PMC5780224

[23] Standl E , Khunti K , Hansen TB , Schnell O . The global epidemics of diabetes in the 21st century: current situation and perspectives. Eur J Prev Cardiol. 2019;26:7–14. 10.1177/2047487319881021 31766915

[24] Chiranthanut N , Teekachunhatean S , Panthong A , Khonsung P , Kanjanapothi D , Lertprasertsuk N . Toxicity evaluation of standardized extract of *Gynostemma pentaphyllum* Makino. J Ethnopharmacol. 2013;149:228–34. 23796877 10.1016/j.jep.2013.06.027

[25] Attawish A , Chivapat S , Phadungpat S , Bansiddhi J , Techadamrongsin Y , Mitrijit O , et al. Chronic toxicity of *Gynostemma pentaphyllum* . Fitoterapia. 2004;75:539–51. 15351107 10.1016/j.fitote.2004.04.010

[26] Park SH , Huh TL , Kim SY , Oh MR , Tirupathi Pichiah PB , Chae SW , et al. Antiobesity effect of *Gynostemma pentaphyllum* extract (actiponin): a randomized, double‐blind, placebo‐controlled trial. Obesity (Silver Spring). 2014;22:63–71. 23804546 10.1002/oby.20539

[27] Kim YH , Kim SM , Lee JK , Jo SK , Kim HJ , Cha KM , et al. Efficacy of *Gynostemma pentaphyllum* extract in anti‐obesity therapy. Rec Nat Prod. 2019. 116–28.

[28] Choi EK , Won YH , Kim SY , Noh SO , Park SH , Jung SJ , et al. Supplementation with extract of *Gynostemma pentaphyllum* leaves reduces anxiety in healthy subjects with chronic psychological stress: a randomized, double‐blind, placebo‐controlled clinical trial. Phytomedicine. 2019;52:198–205. 30599899 10.1016/j.phymed.2018.05.002

[29] Blaak E . Gender differences in fat metabolism. Curr Opin Clin Nutr Metab Care. 2001;4:499–502. 11706283 10.1097/00075197-200111000-00006

[30] Karastergiou K , Smith SR , Greenberg AS , Fried SK . Sex differences in human adipose tissues – the biology of pear shape. Biol Sex Differ. 2012;3:13. 22651247 10.1186/2042-6410-3-13 PMC3411490

[31] Li G , Wu W , Zhang X , Huang Y , Wen Y , Li X , et al. Serum levels of tumor necrosis factor alpha in patients with IgA nephropathy are closely associated with disease severity. BMC Nephrol. 2018;19:326. 30428849 10.1186/s12882-018-1069-0 PMC6236996

[32] Jang M , Park R , Kim H , Namkoong S , Jo D , Huh YH , et al. AMPK contributes to autophagosome maturation and lysosomal fusion. Sci Rep. 2018;8:12637. 30140075 10.1038/s41598-018-30977-7 PMC6107659

[33] Weisberg SP , McCann D , Desai M , Rosenbaum M , Leibel RL , Ferrante AW Jr . Obesity is associated with macrophage accumulation in adipose tissue. J Clin Invest. 2003;112:1796–808. 14679176 10.1172/JCI19246 PMC296995

[34] Xu H , Barnes GT , Yang Q , Tan G , Yang D , Chou CJ , et al. Chronic inflammation in fat plays a crucial role in the development of obesity‐related insulin resistance. J Clin Invest. 2003;112:1821–30. 14679177 10.1172/JCI19451 PMC296998

[35] Fain JN , Bahouth SW , Madan AK . TNFalpha release by the nonfat cells of human adipose tissue. Int J Obes Relat Metab Disord. 2004;28:616–22. 14770194 10.1038/sj.ijo.0802594

[36] Lumeng CN , Bodzin JL , Saltiel AR . Obesity induces a phenotypic switch in adipose tissue macrophage polarization. J Clin Invest. 2007;117:175–84. 17200717 10.1172/JCI29881 PMC1716210

[37] Coenen KR , Gruen ML , Chait A , Hasty AH . Diet‐induced increases in adiposity, but not plasma lipids, promote macrophage infiltration into white adipose tissue. Diabetes. 2007;56:564–73. 17327423 10.2337/db06-1375

[38] GBD 2013 DALYS and HALE Collaborators , Murray CJ , Barber RM , Foreman KJ , Abbasoglu Ozgoren A , Abd‐Allah F , et al. Global, regional, and national disability‐adjusted life years (DALYs) for 306 diseases and injuries and healthy life expectancy (HALE) for 188 countries, 1990‐2013: quantifying the epidemiological transition. Lancet. 2015;386:2145–91. 26321261 10.1016/S0140-6736(15)61340-X PMC4673910

[39] van der Windt DJ , Sud V , Zhang H , Tsung A , Huang H . The effects of physical exercise on fatty liver disease. Gene Expr. 2018;18:89–101. 29212576 10.3727/105221617X15124844266408 PMC5954622

[40] Flores‐Toro JA , Go KL , Leeuwenburgh C , Kim JS . Autophagy in the liver: cell's cannibalism and beyond. Arch Pharm Res. 2016;39:1050–61. 27515049 10.1007/s12272-016-0807-8 PMC5007189

[41] Mihaylova MM , Shaw RJ . The AMPK signalling pathway coordinates cell growth, autophagy and metabolism. Nat Cell Biol. 2011;13:1016–23. 21892142 10.1038/ncb2329 PMC3249400

[42] Tarantino G , Citro V , Capone D . Nonalcoholic fatty liver disease: a challenge from mechanisms to therapy. J Clin Med. 2019;9:9. 10.3390/jcm9010015 PMC7019297 31861591

[43] Hong M , Cai Z , Song L , Liu Y , Wang Q , Feng X . *Gynostemma pentaphyllum* attenuates the progression of nonalcoholic fatty liver disease in mice: a biomedical investigation integrated with In silico assay. Evid Based Complement Alternat Med. 2018;2018:8384631. 29743925 10.1155/2018/8384631 PMC5884411

[44] Bae UJ , Park EO , Park J , Jung SJ , Ham H , Yu KW , et al. Gypenoside UL4‐rich *Gynostemma pentaphyllum* extract exerts a hepatoprotective effect on diet‐induced nonalcoholic fatty liver disease. Am J Chin Med. 2018;46:1315–32. 30180767 10.1142/S0192415X18500696

[45] Shen SH , Zhong TY , Peng C , Fang J , Lv B . Structural modulation of gut microbiota during alleviation of non‐alcoholic fatty liver disease with *Gynostemma pentaphyllum* in rats. BMC Complement Med Ther. 2020;20:34. 32024509 10.1186/s12906-020-2835-7 PMC7076883

[46] Chou SC , Chen KW , Hwang JS , Lu WT , Chu YY , Lin JD , et al. The add‐on effects of *Gynostemma pentaphyllum* on nonalcoholic fatty liver disease. Altern Ther Health Med. 2006;12:34–9. 16708768

